# Reciprocal positive regulation between Cx26 and PI3K/Akt pathway confers acquired gefitinib resistance in NSCLC cells via GJIC-independent induction of EMT

**DOI:** 10.1038/cddis.2015.197

**Published:** 2015-07-23

**Authors:** J Yang, G Qin, M Luo, J Chen, Q Zhang, L Li, L Pan, S Qin

**Affiliations:** 1 Department of Pharmacology, School of Pharmacy, Guangxi Medical University, 22 Shuangyong Road, Nanning 530021, Guangxi, China; 2 Department of Hepatobiliary Surgery, Affiliated Cancer Hospital, Guangxi Medical University, 71 Hedi Road, Nanning 530021, Guangxi, China; 3 Division of Pulmonary, Department of Medicine, Allergy and Critical Care, Lung Biology Laboratory, Columbia University Medical Center, New York, NY 10032, USA; 4 Nephrology Division, The First Affiliated Hospital, Guangxi Medical University, 6 Shuangyong Road, Nanning 530021, Guangxi, China; 5 Department of Respiratory Medicine, The First Affiliated Hospital, Guangxi Medical University, 6 Shuangyong Road, Nanning 530021, Guangxi, China

## Abstract

Gefitinib efficiency in non-small-cell lung cancer (NSCLC) therapy is limited due to development of drug resistance. The molecular mechanisms of gefitinib resistance remain still unclear. In this study, we first found that connexin 26 (Cx26) is the predominant Cx isoform expressed in various NSCLC cell lines. Then, two gefitinib-resistant (GR) NSCLC cell lines, HCC827 GR and PC9 GR, from their parental cells were established. In these GR cells, the results showed that gefitinib resistance correlated with changes in cellular EMT phenotypes and upregulation of Cx26. Cx26 was detected to be accumulated in the cytoplasm and failed to establish functional gap-junctional intercellular communication (GJIC) either in GR cells or their parental cells. Ectopic expression of GJIC-deficient chimeric Cx26 was sufficient to induce EMT and gefitinib insensitivity in HCC827 and PC9 cells, while knockdown of Cx26 reversed EMT and gefitinib resistance in their GR cells both *in vitro* and *in vivo*. Furthermore, Cx26 overexpression could activate PI3K/Akt signaling in these cells. Cx26-mediated EMT and gefitinib resistance were significantly blocked by inhibition of PI3K/Akt pathway. Specifically, inhibition of the constitutive activation of PI3K/Akt pathway substantially suppressed Cx26 expression, and Cx26 was confirmed to functionally interplay with PI3K/Akt signaling to promote EMT and gefitinib resistance in NSCLC cells. In conclusion, the reciprocal positive regulation between Cx26 and PI3K/Akt signaling contributes to acquired gefitinib resistance in NSCLC cells by promoting EMT via a GJIC-independent manner.

Lung cancer, of which non-small-cell lung cancer (NSCLC) is the most common form, remains the leading cause of cancer-related deaths worldwide.^1^ Currently, gefitinib, as the first epidermal growth factor receptor (EGFR) tyrosine kinase inhibitor (TKI), is one of the most accepted therapies against NSCLC carrying EGFR mutations. However, almost all NSCLC patients who initially respond well to EGFR-TKIs eventually develop acquired resistance.^2^ Development of effective therapeutic interventions to overcome gefitinib resistance is an urgent need.

Epithelial-mesenchymal transition (EMT), during which cancer cells lose epithelial markers such as E-cadherin but gain mesenchymal markers such as vimentin, is known to be deeply involved in cancer progression and chemotherapy resistance. Specially in NSCLC, EMT plays pivotal roles in the acquired resistance to EGFR-TKIs such as gefitinib.^3, 4^ For example, restoring E-cadherin expression or silencing EMT regulator Slug increases gefitinib sensitivity in NSCLC cells with a mesenchymal phenotype.^5, 6^ Accumulating evidences indicate that constitutively activation of the phosphoinositide 3-kinase (PI3K)/Akt signaling is a central feature of EMT in many cancers including NSCLC.^7, 8^ However, the exact mechanism for the acquired gefitinib resistance of NSCLC remains unclear.

Connexins (Cxs) are a family of transmembrane proteins, which compose the intercellular gap junctions between the neighboring cells.^9^ Gap junctions directly connect the cytoplasms of adjacent cells, thereby mediating direct exchange of signaling molecules smaller than 1 kDa, such as ions, small metabolites, and second messengers. This process is termed gap-junctional intercellular communication (GJIC). Cx expression and/or GJIC are frequently reduced or loss in malignant cell lines and cancers, while restoration of Cx expression and/or GJIC retarded tumor growth and increased cytotoxicities of chemotherapeutics such as cisplatin and docetaxel.^10, 11, 12, 13^ Therefore, Cxs have long been deemed tumor suppressors. However, increasing new observations were apparently contradicting the 'dogma' and became clear that Cxs and GJIC also contribute to cancer progression and chemoresistance. For example, Cx32 expression was detected in breast cancer and significantly increased in lymph node metastases compared with primary tumors, suggesting Cx32 may be a sign of more malignant phenotype of breast cancer.^14^ Besides, cytoplasmic accumulation of Cx32 exerted favorable effects for hepatocellular carcinoma (HCC) progression including invasion and metastasis by Cx linked, but GJIC-independent mechanism.^15^ Recently, Gielen *et al.*^16^ reported that increasing the level of Cx43 confers temozolomide resistance in human glioma cells whereas knockdown of Cx43 sensitizes them to temozolomide treatment via both GJIC-dependent and -independent mechanisms.

Up to now, there are ~21 isoforms of Cxs that distribute in almost all human organs in tissue-specific patterns.^17^ Cx26, one of the most common isoforms of Cxs, is predominantly expressed in lung tissue.^18, 19^ Despite Cx26 has been considered as a potential tumor suppressor or chemotherapy sensitizer in some types of tumors,^20, 21^ Ito *et al.*^22^ found that Cx26 helps lung squamous cell carcinoma (SCC, one histological type of NSCLC), acquire aggressive phenotypes, lymph node metastasis, and poor prognosis, indicating that a potential role of Cx26 on the malignant development of SCC. However, the roles of Cx26 and its derived GJIC in the development of gefitinib resistance in NSCLC have not been explored.

In this study, to clarify the potential role of Cx26 and its derived GJIC in gefitinib resistance in NSCLC, we first surveyed the expression of four major Cxs in different gefitinib-sensitive NSCLC cell lines and found a positive correlation between high level of Cx26 and gefitinib insensitivity in NSCLC cells. Such an association was further confirmed in established gefitinib-resistant (GR) HCC827 and PC9 cell lines both *in vitro* and *in vivo*. Importantly, we find a positive mutual regulation between Cx26 and PI3K/Akt pathway, which confers acquired gefitinib resistance in NSCLC cells by GJIC-independent induction of EMT.

## Results

### Cx26 upregulation is correlated with gefitinib insensitivity of human NSCLC cells

**Figure 1:**
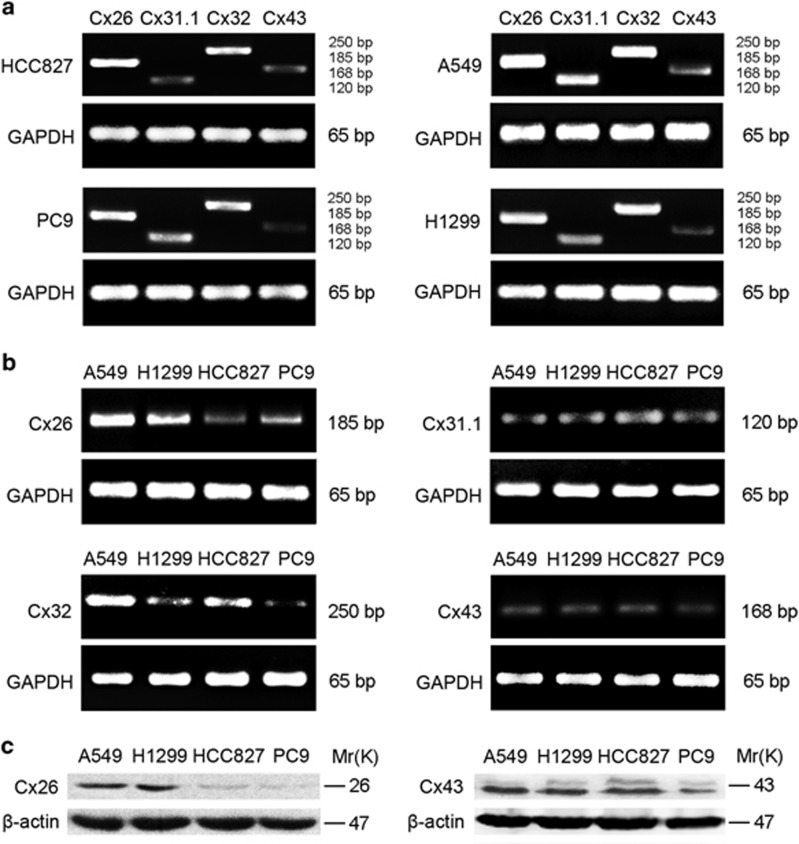
Increased Cx26 is positively correlated with gefitinib resistance in NSCLC cells. (**a**) Differential expression of Cx26, Cx31.1, Cx32, and Cx43 in different gefitinib-sensitive NSCLC cell lines was determined by RT-PCR. (**b** and **c**) High level of Cx26 in gefitinib-insensitive A549 and H1299 cells than that in gefitinib-sensitive HCC827 and PC9 cells was detected by RT-PCR and western blotting. GAPDH or *β*-actin was used as internal loading control

We first performed RT-PCR and western blotting to determine Cx expression phenotype in different gefitinib-sensitive NSCLC cell lines. As shown in Figures 1a–c, four major Cx isoforms, Cx26, Cx32, Cx31.1, and Cx43, were differentially expressed in four NSCLC cell lines (HCC827, PC9, A549, and H1299). In particular, Cx26 was the predominant Cx isoform expressed in various NSCLC cell lines. Moreover, the level of Cx26 was markedly higher in gefitinib-insensitive NSCLC cell lines (A549 and H1299) than that in gefitinib-sensitive NSCLC cell lines (HCC827 and PC9). These results suggest that Cx26 may be positively correlated with gefitinib insensitivity in NSCLC cells.

### GR HCC827 and PC9 cell lines were established with acquired EMT characteristics and elevated Cx26 expression

**Figure 2:**
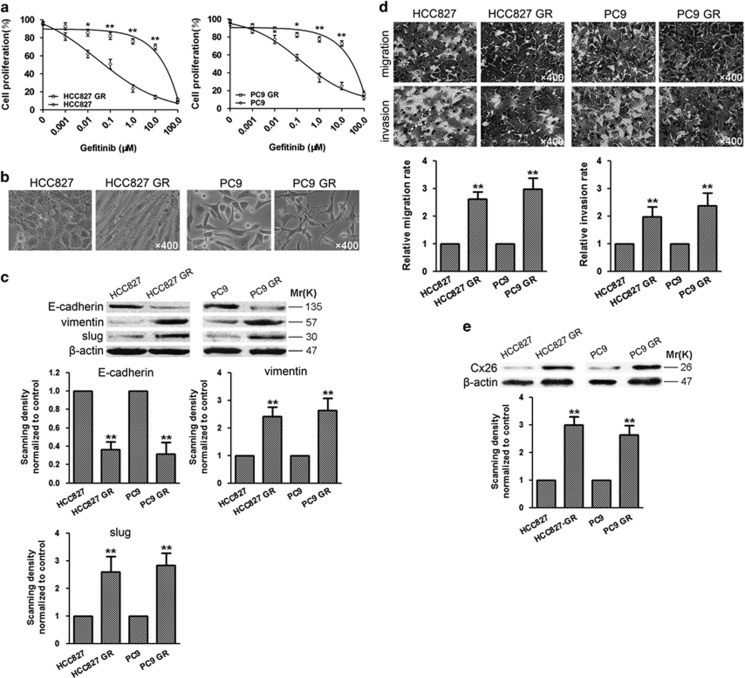
Gefitinib-resistant (GR) HCC827 and PC9 cell lines were established with acquired EMT characteristics and elevated Cx26 expression. (**a**) Comparison of gefitinib sensitivity between parental HCC827, PC9 cells, and their GR cells. Cell proliferation was measured by MTT assay. Data are presented as mean±S.D. from four independent experiments. *P<0.05 and **P<0.01 for GR cells *versus* parental cells. (**b**) Morphological changes of HCC827 GR and PC9 GR cells. Original magnification, × 400. (**c**) Western blot analysis of EMT-associated proteins. Bar graphs are derived from densitometric scanning of the blots. Error bars are mean±S.D. from three independent experiments. **P<0.01 for GR cells *versus* parental cells. (**d**) Migratory and invasive abilities of HCC827 GR, PC9 GR cells, and their parental cells were determined by Transwell assays. Error bars are mean±S.D. from four independent experiments. **P<0.01 for GR cells *versus* parental cells. Original magnification, × 400. (**e**) Western blot analysis of Cx26 protein expression in HCC827 GR, PC9 GR cells, and their parental cells. Bar graphs are derived from densitometric scanning of the blots. Error bars are mean±S.D. from four independent experiments. **P<0.01 for GR cells *versus* parental cells

To explore the mechanism of acquired gefitinib resistance of NSCLC, we generated two acquired GR NSCLC cell lines, HCC827 GR and PC9 GR, from their parental cells by continuous exposure to gefitinib starting at 0.001 *μ*M and increasing in a stepwise manner to 1 *μ*M. As shown in Figure 2a, gefitinib showed less cytotoxicity in established HCC827 GR and PC9 GR cells than that in their parental cells with IC50 of 12.17±0.18 *μ*M *versus* 0.06±0.11 *μ*M and 16.51±0.17 *μ*M *versus* 0.25±0.07 *μ*M, respectively.

Moreover, HCC827 GR and PC9 GR cells exhibited scattered, elongated, and mesenchymal-like morphology, while their parental HCC827 and PC9 cells showed rounded shape, typical of epithelial cobblestone appearance (Figure 2b). Consistently, the expression of epithelial marker E-cadherin was greatly reduced, whereas the level of mesenchymal marker vimentin and slug was significantly elevated in HCC827 GR and PC9 GR cells (Figure 2c). A key feature of cancer cells undergoing EMT is enhanced migratory and invasive potential. As shown in Figure 2d, mobility and invasive capability of HCC827 GR and PC9 GR cells were significantly increased by 2.6- or 3.0-fold and 2.0- or 2.4-fold compared with their parental cells, respectively. Moreover, the levels of Cx26 were increased in HCC827 GR and PC9 GR cells (Figure 2e). These results suggest a potential role of Cx26 in the acquisition of EMT and acquired gefitinib resistance of NSCLC cells.

### Cx26 induces acquired gefitinib resistance in NSCLC cells via GJIC-independent manner

**Figure 3:**
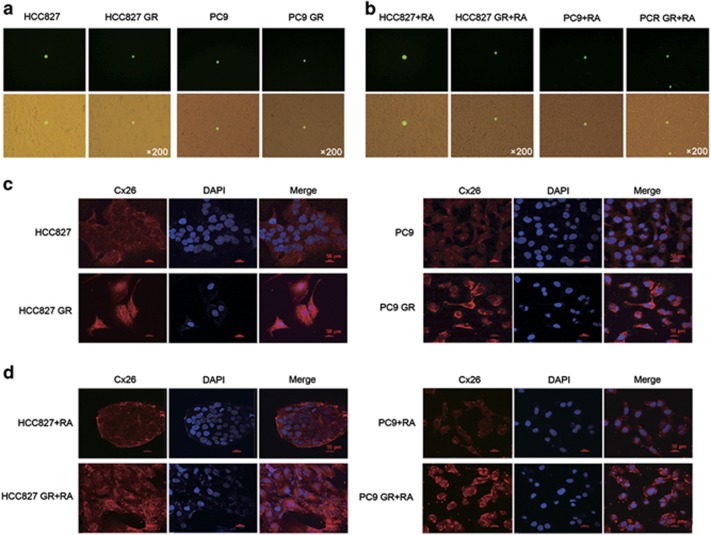
Cx26 induces acquired gefitinib resistance in NSCLC cells via GJIC-independent manner. (**a**) Functional GJIC was detected by parachute assay and no detectable GJIC was found in HCC827 GR, PC9 GR, and their parental cells. Top: fluorescence images. Bottom: overlaid the corresponding phase-contrast images. Original magnification, × 200. (**b**) No enhancement of GJIC in these cells incubated with 10, 20, and 40 *μ*M of RA (a well-defined GJIC enhancer) for 4, 8, 12, 24, and 48 h, respectively. Top: fluorescence images. Bottom: overlaid the corresponding phase-contrast images. Original magnification, × 200. (**c** and **d**) Immunofluorescence staining of the cellular localization of Cx26 with or without RA treatment. All scare bars represent 50 *μ*m

Cxs have long been believed to regulate tumor development during carcinogenesis by exerting GJIC. Therefore, we next examined whether GJIC was involved in Cx26-induced EMT and acquired gefitinib resistance of NSCLC cells. First, GJIC in primarily human foreskin fibroblasts (HFFs) as positive control was confirmed, and treatment of these cells with RA (a well-defined GJIC enhancer) significantly enhanced GJIC among these cells. As shown in Figure 3a, no detectable GJIC was found in HCC827, PC9, and their GR cells. To exclude the involvement of undetectable GJIC in these cells, GJIC was further measured in the presence of 10, 20, and 40 *μ*M of RA for 4, 8, 12, 24, and 48 h, respectively. The result showed no enhancement of GJIC in these cells incubated with RA (Figure 3b). These observations suggest that Cx26 is not functional as a component of gap junctions in these cells. Furthermore, we assessed the subcellular localization of Cx26 in these cells. Figure 3c clearly showed that Cx26 protein accumulated in the cytoplasm of HCC827, PC9 cells, and their GR cells. Although, incubated with the GJIC enhancer RA, Cx26 was still retained in the cytoplasm (Figure 3d). These results indicate that Cx26 cannot form functional gap junctions between these cells due to the absence of integration into plasma membrane, confirming that GJIC is not implicated in the Cx26-mediated EMT and acquired gefitinib resistance in NSCLC cells.

**Figure 4:**
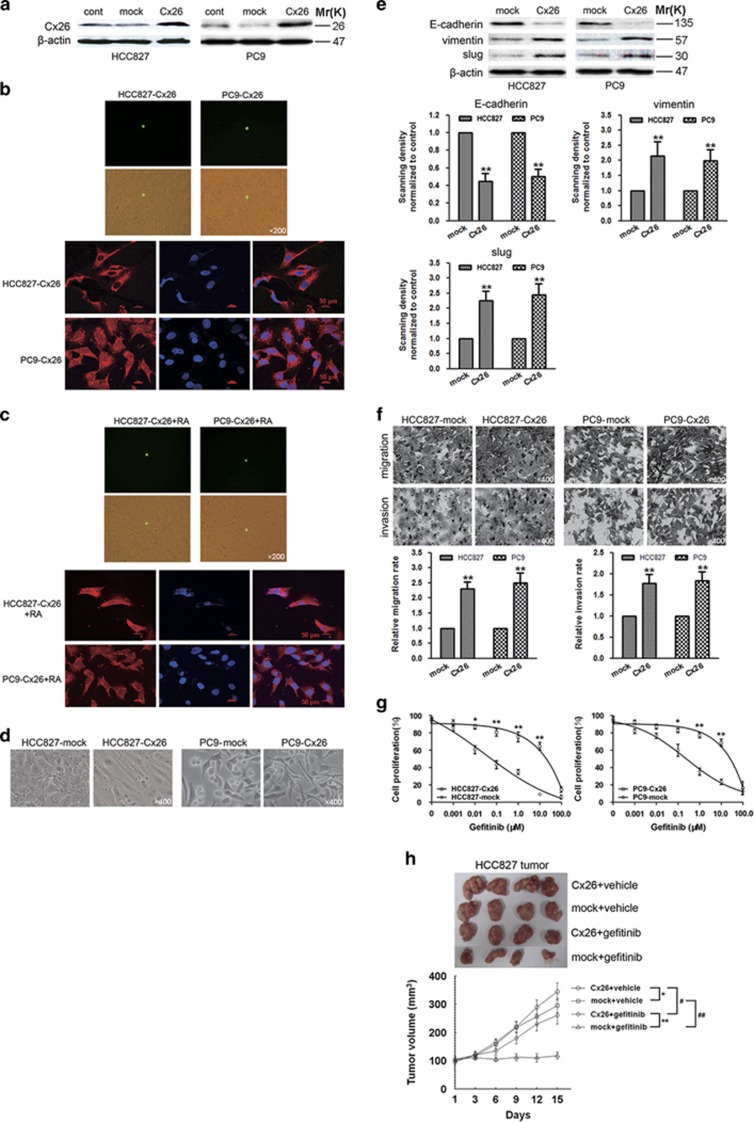
Overexpression of Cx26 induces EMT and gefitinib resistance in HCC827 and PC9 cells via GJIC-independent manner. (**a**) Western blotting showed the successful lentiviral infections of GJIC-deficient chimeric Cx26. (**b** and **c**) Parachute assay and immunofluorescence staining of Cx26-overexpressing HCC827 and PC9 cells with or without RA treatment. Original magnification, × 200. All scare bars of immunofluorescence pictures represent 50 *μ*m. (**d**) Morphological changes of Cx26-overexpressing HCC827 and PC9 cells and their mock-infected counterparts. Original magnification, × 400. (**e**) Effect of Cx26 overexpression on the expression of E-cadherin, vimentin, and slug was evaluated by western blotting in HCC827 and PC9 cells. Bar graphs are derived from densitometric scanning of the blots. Error bars are mean±S.D. from four independent experiments. **P<0.01 *versus* mock-infected cells. (**f**) Effect of Cx26 overexpression on the migratory and invasive abilities of HCC827 and PC9 cells was measured by Transwell assay. Error bars are mean±S.D. from four independent experiments. **P<0.01 *versus* mock-infected cells. Original magnification, × 400. (**g**) Effect of Cx26 overexpression on gefitinib efficacy in HCC827 and PC9 cells was detected by MTT assay. Error bars are mean±S.D. from four independent experiments, *P<0.05 and **P<0.01 *versus* mock-infected cells. (**h**) HCC827 cells stably expressing Cx26 or its mock control were transplanted into athymic mice (*n*=6 per group), respectively. Tumor size was measured every 3 days for indicated period. The representative tumors and growth curves of tumor are shown. Error bars are mean±S.D. *P<0.05 and **P<0.01 *versus* mock-infected groups. ^#^*P*<0.05 and ^##^*P*<0.01 *versus* vehicle treatment groups

To explore the role of Cx26 *per se* in the regulation of EMT and acquired gefitinib resistance in NSCLC, we engineered GJIC-deficient HCC827 and PC9 cells stably expressing chimeric Cx26 with the green fluorescent protein (GFP) fused to the amino-terminal (Figure 4a). Characterization of this chimeric protein revealed that Cx26 accumulated in the cytoplasm and failed to establish functional GJIC (Figure 4b). After incubation with RA, Cx26 was still retained in the cytoplasm with no detectable GJIC (Figure 4c). Despite lack of GJIC, overexpression of Cx26 *per se* was sufficient to induce elongated mesenchymal-like morphology transition (Figure 4d), consistent with decreased expression of E-cadherin while increased expression of vimentin and slug (Figure 4e), and enhanced migratory and invasive potential of HCC827 and PC9 cells (Figure 4f). Furthermore, Cx26 overexpression exerted obvious gefitinib insensitivity in these cells (Figure 4g). Besides, the *in vivo* data showed that administration of gefitinib (100 mg/kg per day, gavaged orally) led to more significant inhibition of HCC827-mock tumor xenografts than HCC827-Cx26 xenografts, compared with vehicle groups (Figure 4h). These results reinforce the GJIC-independent role of Cx26 in the promotion of EMT and gefitinib resistance in NSCLC.

**Figure 5:**
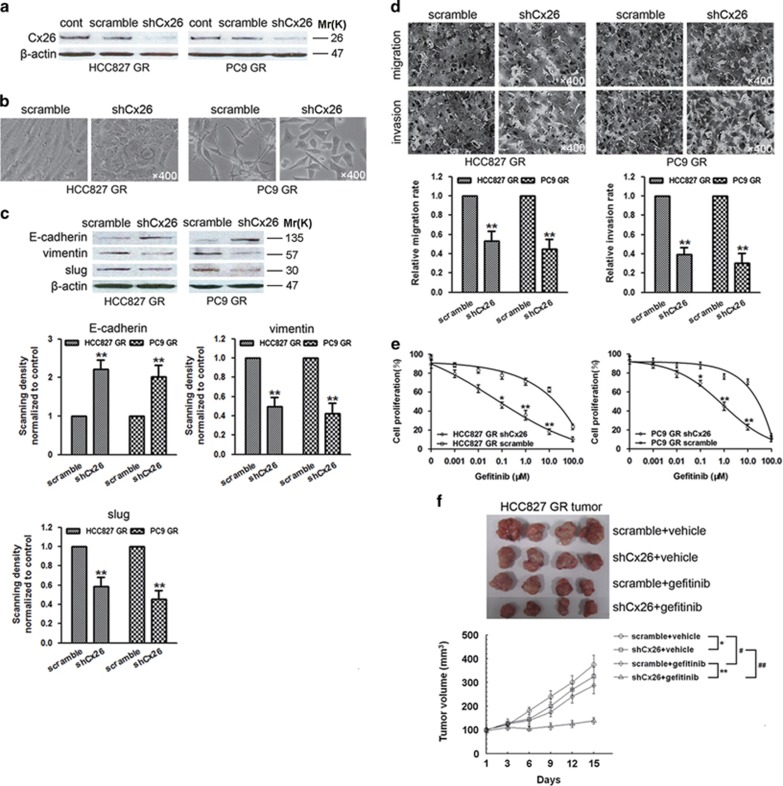
Knockdown of Cx26 reverses EMT and gefitinib resistance in HCC827 GR and PC9 GR cells. (**a**) The expression of Cx26 was determined by western blotting after lentiviral infections of HCC827 GR and PC9 GR cells with shRNA against Cx26 (shCx26) or scramble shRNA. (**b**) Morphological changes of HCC827 GR and PC9 GR cells harboring shCx26 or scramble shRNA were evaluated by phase-contrast microscopy. Original magnification, × 400. (**c**) Effect of Cx26 knockdown on the expression of E-cadherin, vimentin, and slug was analyzed by western blotting in HCC827 GR and PC9 GR cells. Bar graphs are derived from densitometric scanning of the blots. Error bars are mean±S.D. from four independent experiments. **P<0.01 *versus* scramble-infected cells. (**d**) Effect of Cx26 knockdown on the migratory and invasive abilities of HCC827 GR and PC9 GR cells was assessed by Transwell assay. Error bars are mean±S.D. from five independent experiments. **P<0.01 *versus* scramble-infected cells. Original magnification, × 400. (**e**) Effect of Cx26 knockdown on gefitinib efficacy in HCC827 GR and PC9 GR cells was detected by MTT assay. Error bars are mean±S.D. from five independent experiments, *P<0.05 and **P<0.01 *versus* scramble-infected cells. (**f**) HCC827 GR cells stably transfecting shCx26 or its scramble shRNA were transplanted into athymic mice (*n*=6 per group), respectively. Tumor size was measured every 3 days for indicated period. The representative tumors and growth curves of tumor are shown. Error bars are mean±S.D. *P<0.05 and **P<0.01 *versus* scramble-infected groups. ^#^*P*<0.05 and ^##^*P*<0.01 *versus* vehicle treatment groups

To further confirm the effect of Cx26 on EMT and gefitinib resistance in NSCLC, we transducted Cx26 short hairpin RNA (shRNA, shCx26) or scramble shRNA into HCC827 GR and PC9 GR cells (Figure 5a). Knockdown of Cx26 expression significantly restored the rounded epithelial-like appearance (Figure 5b), enhanced E-cadherin expression while reduced vimentin and slug expression (Figure 5c), and meanwhile strongly inhibited migratory and invasive potential of HCC827 GR and PC9 GR cells (Figure 5d). Moreover, gefitinib efficacy was substantially increased in shCx26-transduced HCC827 GR and PC9 GR cells (Figure 5e). The capability of Cx26 depletion to restore gefitinib sensitivity of NSCLC was also observed in *in vivo* tumor model. As shown in Figure 5f, administration of gefitinib (100 mg/kg per day, gavaged orally) triggered more dramatic regression of shCx26-transduced HCC827 GR tumor xenografts than scramble HCC827 GR xenografts, compared with vehicle groups.

Taken together, these results indicate that Cx26 *per se*, but not the extent of GJIC, corresponds to acquired gefitinib resistance in NSCLC cells via induction of EMT both *in vitro* and *in vivo*.

### Reciprocal positive regulation between Cx26 and PI3K/Akt pathway is involved in Cx26-mediated EMT and gefitinib resistance of NSCLC cells

**Figure 6:**
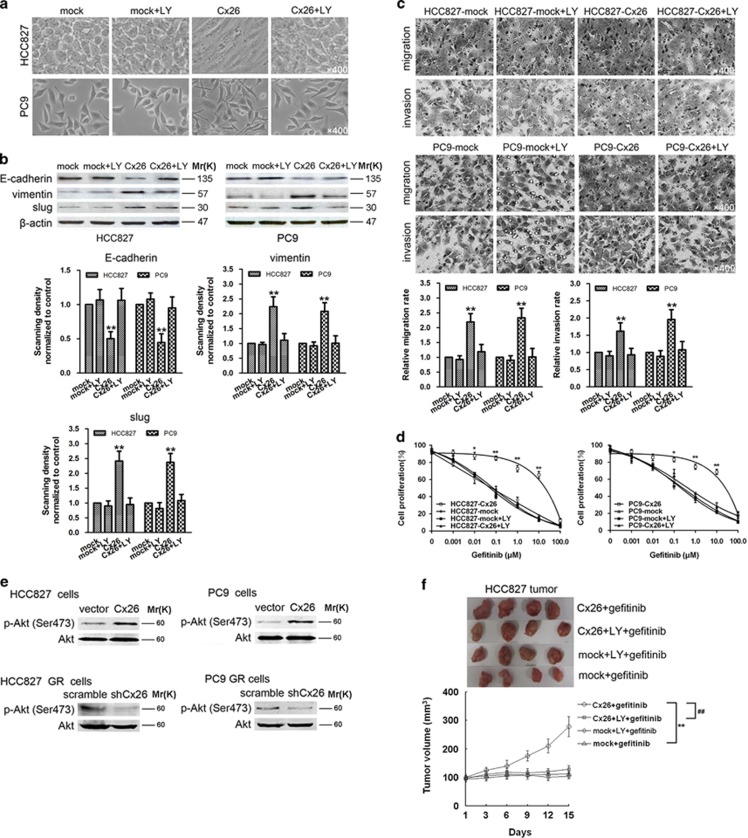
Upregulation of Cx26 induces EMT and gefitinib resistance via PI3K/Akt-dependent pathway. (**a**) Effect of the PI3K/Akt pathway-specific inhibitor LY294002 (LY) on Cx26 overexpression-induced morphological changes in HCC827 and PC9 cells. Original magnification, × 400. (**b**) Effects of LY294002 on Cx26 overexpression-induced changes in the expression of E-cadherin, vimentin, and slug in HCC827 and PC9 cells. Bar graphs are derived from densitometric scanning of the blots. Error bars are mean±S.D. from three independent experiments. **P<0.01 *versus* mock-infected cells. (**c** and **d**) Effects of LY294002 on Cx26 overexpression-mediated increased migration and invasion and decreased gefitinib efficacy in HCC827 and PC9 cells. Error bars are mean±S.D. from five (**c**) or four (**d**) independent experiments, *P<0.05 and **P<0.01 *versus* mock-infected cells. Original magnification, × 400. (**e**) Effect of Cx26 overexpression or depletion on Akt activity was determined by western blot. (**f**) Cx26-overexpressing HCC827 and its mock control tumor-bearing animals were treated with LY294002 (25 mg/kg, twice a week, i.p.) and/or gefitinib (100 mg/kg per day, gavaged orally). Tumor size was measured every 3 days for indicated period. The representative tumors and growth curves of tumor are shown. Error bars are mean±S.D. **P<0.01 *versus* mock-infected group. ^##^*P*<0.01 *versus* LY treatment group

Based on the aforementioned observations, we became interested in exploring the molecular mechanism underlying the GJIC-independent role of Cx26 in the stated effects. PI3K/Akt pathway is known to play a prominent role in driving EMT and drug resistance in cancers.^23^ It has been reported that activation of PI3K/Akt signaling could directly increase Cx43 phosphorylation^24^ and Cx43 also could contribute to activation of PI3K/Akt signaling.^25^ Therefore, we sought to determine whether there exists a reciprocal activation between Cx26 and PI3K/Akt pathway in promoting EMT and acquired gefitinib resistance of NSCLC cells. As shown in Figures 6a–d, treatment of Cx26-overexpressing HCC827 and PC9 cells with a specific PI3K/Akt pathway inhibitor LY294002 (25 *μ*M) for 24 h could apparently antagonize the facilitating effects of Cx26 on EMT and gefitinib resistance of NSCLC cells. However, LY294002 treatment only had little effect on cell invasion and migration, as well as gefitinib efficacy *in vitro*. Consistent results were obtained from these cells treated with another selective PI3K/Akt pathway inhibitor wortmannin (10 *μ*M) for 4 h (data not shown). Moreover, Cx26 overexpression significantly activated PI3K/Akt pathway as represented by elevated Akt phosphorylation in HCC827 and PC9 cells, while Cx26 depletion caused reduced PI3K/Akt activity in HCC827 GR and PC9 GR cells (Figure 6e). *In vivo* studies showed that treatment with LY294002 (25 mg/kg, twice a week, i.p.) induced marked tumor regression of Cx26-overexpressing group to the level comparable to that of mock control group (Figure 6f). Together, these findings suggest that activation of PI3K/Akt pathway is sufficient to account for Cx26-promoted EMT and gefitinib resistance in NSCLC cells.

**Figure 7:**
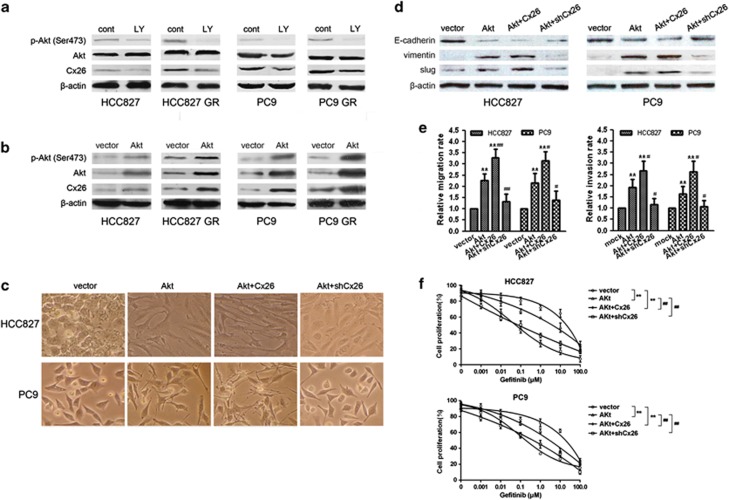
Cx26 and PI3K/Akt pathway functionally interplay to promote EMT and gefitinib resistance in NSCLC cells. (**a** and **b**) Effect of LY294002 or Akt overexpression on Cx26 expression in HCC827, PC9, and their GR cells was determined by western blotting. (**c**) Effects of Akt overexpression alone or combined with Cx26 overexpression or Cx26 depletion on cell morphology changes in HCC827 and PC9 cells. Original magnification, × 400. (**d**–**f**) Effects of Akt overexpression alone or combined with Cx26 overexpression or Cx26 depletion on the expression of EMT markers (E-cadherin, vimentin, and slug), cell migration, and invasion, as well as cell sensitivity to gefitinib in HCC827 and PC9 cells, respectively. Error bars are mean±S.D. from four independent experiments, **P<0.01 *versus* vector group. ^#^*P*<0.05 and ^##^*P*<0.01 *versus* Akt-overexpressing group

PI3K/Akt pathway is constitutively activated in various cancers including NSCLC.^26, 27^ Thus, we were interested in whether Akt activation induces Cx26 expression. As shown in Figure 7a, treatment with 25 *μ*M LY294002 for 24 h caused a significant reduced Cx26 expression both in HCC827, PC9 cells, and their GR cells. Moreover, ectopic expression of Akt significantly increased Cx26 expression in these cells (Figure 7b).

In addition, we investigated the biological significance of the mutual positive regulation between Cx26 and PI3K/Akt pathway in EMT and gefitinib resistance of NSCLC cells. As shown in Figures 7c–f, Akt overexpression alone also induced EMT and gefitinib resistance of HCC827 and PC9 cells. Cx26 overexpression strengthened Akt-facilitated EMT and gefitinib resistance, whereas Cx26 depletion rendered impaired Akt-promoted effects in these cells. Collectively, these results indicate that interdependent positive regulation of Cx26 and PI3K/Akt pathway contributes to gefitinib resistance in NSCLC through induction of EMT.

## Discussion

We present here that a reciprocal positive regulation exists between Cx26 and PI3K/Akt signaling, thus providing insights into the molecular mechanism underlying the dysregulation of Cx26 and PI3K/Akt in NSCLC cells. Furthermore, the functional interplay between Cx26 and PI3K/Akt signaling contributes to the acquired gefitinib resistance in NSCLC cells by GJIC-independent induction of EMT.

Cxs are frequently deregulated in cancers from different origins, either by reduction, lack of expression, or upregulation.^28, 29^ In this study, we found that various NSCLC cell lines have high level of Cx26, but moderate level of Cx32 and Cx31.1, and only low level of Cx43. Such aberrant Cx expression is in agreement with accumulating evidences indicating that different Cxs have different facets in cancer chemoresistance. For instance, Yu *et al.*^30^ reported that Cx43 overexpression reversed EMT and cisplatin resistance in cisplatin-resistant NSCLC cell lines. On the contrary, two recent reports showed that Cx43 knockdown could sensitize glioblastoma cells to temozolomide.^16, 31^ Especially for Cx26, its upregulation improved gemcitabine anticancer efficacy in pancreatic cancer cells.^21^ However, in this study, we demonstrate that Cx26 is the predominant Cx isoform expressed in NSCLC cells, and Cx26 upregulation contributes to gefitinib resistance via induction of cell EMT. Together, while these opposing observations underscored the complex role of Cxs in the development of cancer chemoresistance, our results reveal a novel role of Cx26 that implicates in the acquisition of EMT and gefitinib resistance in NSCLC cells.

Cxs have long been believed to regulate chemoresistance by exerting GJIC. Many studies have showed the functional GJIC-dependent enhancing effects of Cx43, Cx37, Cx32, and Cx26 on the toxicity of chemotherapeutic agents in cancer cells.^21, 32, 33, 34^ However, the GJIC-independent effects of Cxs cannot be discarded, as increasing evidences point the facilitating roles of Cxs in tumorigenesis and cancer chemoresistance via GJIC-independent manner. For example, Cx43 could promote the resistance to temozolomide or cisplatin in cancer cells in a GJIC-independent manner.^16, 35^ Moreover, the cytoplasmic Cx32 protein itself, which failed to form GJIC, could facilitate progression of HCC.^15^ In this work, ‘parachute' dye-coupling assay showed no functional GJIC in HCC827 and PC9 cells with low Cx26 expression, and their GR cells with high Cx26 expression. Immunofluorescence staining revealed that Cx26 is aberrantly accumulated in the cytoplasm but not in the normal cell-cell contact areas in these cells. Pharmacological stimulation using RA, a well-defined GJIC enhancer, has no enhancement effects on GJIC in these cells, and could not change the cytoplasmic localization of Cx26. Thus, these results indicate that Cx26 is incapable of forming functional GJIC between NSCLC cells due to the defects in plasma membrane assembly, excluding the possible involvement of GJIC in the Cx26-mediated EMT and acquired gefitinib resistance in NSCLC cells.

Many studies support a role of Cx26 in tumorigenesis that might be independent of GJIC. Cytoplasmic accumulation of Cx26 has been associated with lung metastasis in colorectal cancer^36^ and with poor prognosis in NSCLC and breast carcinoma.^22, 37^ Actually, in the present study, we found that overexpression of chimeric Cx26, which resulted in a significantly increase of Cx26 in the cytoplasm of HCC827 and PC9 cells, was sufficient to induce EMT phenotypes and gefitinib insensitivity *in vitro* and *in vivo*. On the contrary, knockdown of Cx26 reversed EMT and gefitinib resistance in their GR cells and the tumor model. Taken together with the above observations, these results reinforce the GJIC-independent role of Cx26 in the promotion of EMT and gefitinib resistance in NSCLC.

PI3K/Akt pathway-dependent EMT has been shown to contribute to cisplatin resistance in HCC cells^38^ and gefitinib resistance in head and neck SCC cells.^39^ Therefore, in this study, whether EMT and gefitinib resistance in NSCLC cells mediated by Cx26 itself is dependent on PI3K/Akt pathway was determined. We found that inhibition of PI3K/Akt by specific inhibitors LY294002 or wortmannin could reverse EMT and gefitinib resistance in Cx26-overexpressed NSCLC cells. Inhibition of PI3K/Akt also led to tumor regression in Cx26-overexpressed xenografts. Moreover, Cx26 overexpression significantly activated Akt in parental NSCLC cells, while Cx26 depletion reduced PI3K/Akt activity in their GR cells. Consequently, these results indicate that Cx26 contributes to EMT and gefitinib resistance in NSCLC cells mainly through activation of PI3K/Akt pathway.

However, the mechanisms by which Cx26 stimulates PI3K/Akt pathway in NSCLC cells have not been explored. Cx43 has been shown to contribute to the activation of PI3K/Akt signaling possibly as a cofactor of G*β* in cardiomyocytes.^25^ Moreover, a positive correlation between Cx26 expression and insulin-like growth factor receptor I (IGF-IR) has been demonstrated in human colorectal cancer.^40^ IGF-IR upregulation could mediate resistance to EGFR-TKI therapy in primary human glioblastoma cells through continued activation of PI3K/Akt signaling.^41^ These findings combined with ours suggest that the mechanisms for Cx26-stimulated PI3K/Akt pathway are complicated and there may be crosstalk with other signals, such as IGF-IR, to subsequently activate PI3K/Akt pathway.

Interestingly, herein, we also demonstrated that inhibition of PI3K/Akt pathway results in decreased Cx26 expression, whereas overexpression of Akt increases Cx26 expression in NSCLC cells. Supporting these observations was the involvement of activation of PI3K/AKT pathway in TGF-*β*1-induced Cx43 expression.^42^ Besides, activation of PI3K/AKT pathway by shear stress led to increased nuclear accumulation of *β*-catenin, which could bind to the Cx43 promoter and stimulate Cx43 expression.^43^ Therefore, our results demonstrate that there exists a positive feedback regulation between Cx26 expression and PI3K/Akt pathway in NSCLC cells.

In addition, our study showed that overexpression of either Cx26 or Akt alone results in EMT phenotypes and gefitinib resistance in NSCLC cells. Furthermore, Cx26 overexpression enhanced Akt-induced EMT and gefitinib resistance, while Cx26 knockdown led to impaired Akt-mediated effects in these cells. These results indicate that dysfunction of either Cx26 or Akt contributes to acquisition of EMT and gefitinib resistance in NSCLC cells. More importantly, the positive regulatory circuit that mutually reinforces the Cx26 expression and PI3K/Akt activity further augments the EMT and gefitinib resistance in NSCLC cells.

Despite further studies are needed to explore the efficacy of disruption of regulatory network between Cx26 expression and PI3K/Akt pathway in targeted therapy for NSCLC with aberrant Cx26 expression or PI3K/Akt activation, our study shows a positive regulatory loop between Cx26 expression and PI3K/Akt pathway, which confers the acquired gefitinib resistance in NSCLC cells by promoting EMT in a GJIC-independent manner. In conclusion, the Cx26 may be an attractive target for overcoming gefitinib resistance in NSCLC therapy.

## Materials and Methods

### Reagents and antibodies

Gefitinib was provided by AstraZeneca (London, UK) and dissolved in dimethyl sulfoxide (DMSO) at the stock concentration of 10 mM (stored at −20°C) and then diluted in a culture medium before use. Cell culture reagents were obtained from Invitrogen (Carlsbad, CA, USA). Anti-Cx26 antibody was from Sigma-Aldrich (St. Louis, MO, USA). Antibodies against E-cadherin, vimentin, slug, p-Akt (Ser473), Akt, and LY294002 (PI3K inhibitor) were purchased from Cell Signaling Technology (Danvers, MA, USA). All-trans-retinoic acid (RA, GJIC enhancer) was purchased from Merck (San Diego, CA, USA). All other reagents were from Sigma-Aldrich unless stated otherwise.

### Cell lines and cell culture

Human NSCLC cell lines (HCC827, PC9, A549, and H1299) were originally obtained from the ATCC (Manassas, VA, USA). Cells were maintained at 37°C and 5% CO_2_ in RPMI 1640 supplemented with 10% fetal bovine serum (Gibco, Grand Island, NY, USA) and antibiotics (100 units/ml penicillin and 100 *μ*g/ml streptomycin). Two gefitinib-sensitive NSCLC cell lines HCC827 and PC9, both harboring EGFR exon 19 in-frame deletion mutation,^6^ were exposed to increasing concentrations of gefitinib and generated GR cells as reported previously.^2, 44^ The procedures of isolation and culture of HFFs were shown in the Supplementary File.

### RNA interference and overexpression of Cx26

The hU6-MCS-ubiquitin-EGFP-IRES-puromycin lentiviral RNAi vectors (GeneChem, Shanghai, China) containing shRNA against Cx26 and Ubi-MCS-EGFP-IRES-puromycin lentiviral vector (GeneChem) containing chimeric Cx26 where the GFP is tagged to the amino-terminal of Cx26 were constructed as other studies reported previously.^45, 46^ The shRNA with no target gene (scramble) or empty lentiviral vector (mock) was used as control. Cells were infected by lentiviral supernatant plus 5 *μ*g/ml Polybrene (GeneChem) and selected by 10 *μ*g/ml puromycin for 14 days. Thereafter, the resistant clones were pooled and analyzed for Cx26 knockdown or overexpression by western blotting.

### RT-PCR

Total RNA was extracted from cells using TRIzol reagent according to the manufacturer's protocol (Invitrogen). The cDNA was synthesized using iScript reverse transcriptase reagent from 2 *μ*g of total RNA. Cx26, Cx31.1, Cx32, Cx43, and glyceraldehyde-3-phosphate dehydrogenase (GAPDH) detection were carried out with the following primers: 5′-GCTGCAAGAACGTGTGCTA-3′ (sense) and 5′-TGGGTTTTGATCTCCTCGAT-3′ (antisense); 5′-ACCTGGTGAGCAAGAGATGC-3′ (sense) and 5′-CACCCGAAAGGAGGTCGTC-3′ (antisense); 5′-ACCAATTCTTCCCCATCTCC-3′ (sense) and 5′-AAGACGGCCTCAAACAACAG-3′ (antisense); 5′-AGGAGTTCAATCACTTGGCG-3′ (sense) and 5′-GCAGGATTCGGAAAATGAAA-3′ (antisense); and 5′-AGCCACATCGCTCAGACA-3′ (sense) and 5′-GCCCAATACGACCAAATCC-3′ (antisense). The RT-PCR reaction conditions were as follows: stage 1, 95°C for 5 min; stage 2, 30 cycles of 94°C for 45 s, 58°C for 30 s, and 72°C for 45 s; and stage 3, 72°C for 5 min. All the data were normalized relative to the expression of GAPDH mRNA in respective samples.

### Western blot analysis

Western blot protocol was according to our previous report.^47^ The blotted membrane was incubated with primary antibodies at final dilutions ranging between 1/1000 and 1/2000 and then probed with horseradish peroxidase (HRP)–labeled anti-rabbit secondary antibody. Antibody binding was detected by enhanced chemiluminescence (ECL) detection kit (Thermo Fisher Scientific, Waltham, MA, USA) and captured on X-ray film. The densitometry of the bands was quantified by Quantity One software on a GS-800 densitometer (Bio-Rad Laboratories, Hercules, CA, USA).

### MTT assay

As we previously reported,^48^ 3 × 10^4^ cells in 100 *μ*l of complete medium were cultured in 96-well plates and incubated overnight. Then cells were treated with various agents for 96 h. After then, 20 *μ*l of MTT labeling reagent (5 mg/ml) was added to the designated wells, and cells were incubated at 37°C for 4 h. Then, the supernatant was removed and 150 *μ*l DMSO was added to the given wells. After incubation for 15 min at 37°C, the optical density (OD) of plates was read at 570 nm on a micro-ELISA plate reader (Thermo Multiskan MK3, Waltham, MA, USA).

### Cell migration and invasion assay

Cell invasion assay was performed according to our previous study.^49^ The invaded cells were determined as eight high-power fields of cells were counted in each well under an inverted microscope at × 200 magnification. Invasion was presented as the relative invasive rate, which is calculated by the invasive rate of the treated group (number of invaded cells per total cell number) divided by that of the control group. The protocol of the migration assay was the same as that of invasion assay, except that no Matrigel (BD Biosciences, San Jose, CA, USA) was used.

### 'Parachute' dye-coupling assay

Functional GJIC was detected as described by Goldberg *et al.*^50^ Donor and receiver cells were grown to confluence. Donor cells were double-labeled with 5 *μ*mol/l CM-DiI (Life Technologies, Carlsbad, CA, USA), a membrane dye that does not spread to coupled cells, and with 5 *μ*mol/l calcein acetoxymethyl ester, which is converted intracellularly into the gap junction-permeable dye calcein. The donor cells were then trypsinized and seeded onto the receiver cells at 1 : 150 ratio. The donor cells were allowed to attach to the monolayer of receiver cells and form GJIC for 4 h at 37°C, and then examined with a fluorescence microscope (Nikon, TF-1, Tokyo, Japan). The average number of receiver cells containing dye per donor cell was considered as a measure of the degree of GJIC. For the studies involving GJIC enhancer, the donor and receiver cells were exposed to RA (10, 20, and 40 *μ*M) for indicated time (4, 8, 12, 24, and 48 h) in which the donor cells were plated onto the receiver cell monolayer.

### Immunofluorescence

The cells were grown on coverslips for 24 h and then fixed in cold methanol for 10 min. Cells were blocked in 2% bovine serum albumin for 30 min at room temperature and incubated with goat anti-Cx26 primary antibody (diluted 1 : 50, Santa Cruz, Dallas, TX, USA) overnight at 4°C. The cells were then incubated with FITC-conjugated donkey anti-goat secondary antibody (diluted 1 : 200, Abcam, Cambridge, MA, USA) for 1 h at room temperature followed by counterstaining with 4′,6-diamidino-2-phenylindole (DAPI). Images were acquired on a confocal laser scanning fluorescence microscope (Nikon A1) and analyzed using the NIS-Elements software (Nikon Corporation, Tokyo, Japan).

### Xenograft studies in athymic mice

All animal experiments were approved by the Institutional Animal Care and Use Committee of Guangxi Medical University, China. Five- to 6-week-old female nu/nu athymic mice were obtained from the Laboratory Animal Center of Guangxi Medical University. Cells were injected subcutaneously (1 × 10^7^ cells per 200 *μ*l PBS per mouse) into the right flank of these mice. When tumor volumes (calculated as (length × width^2^)/2) reached ~100 mm^3^, as measured by calipers every 3 days, mice were randomly allocated into groups of six animals to receive gefitinib (100 mg/kg per day)^51^ or vehicle by oral gavage, or a combination of gefitinib (100 mg/kg per day) and LY294002 (25 mg/kg, twice a week, i.p.).^52^ All mice were killed on day 15 after their tumor size had been measured.

### Statistical analysis

Data were presented as means±S.D. and were statistically analyzed with unpaired Student's *t*-test at a significance level *P<0.05 and **P<0.01 using Sigma Plot 10.0 software (Jandel Scientific, San Rafael, CA, USA).

## Abbreviations

Cx: connexin
Cx26: connexin 26
NSCLC: non-small-cell lung cancer
EMT: epithelial-mesenchymal transition
GJIC: gap-junctional intercellular communication
PI3K: phosphoinositide 3-kinase
Akt: protein kinase B
