# Supplementary material for: Reciprocal positive regulation between Cx26 and PI3K/Akt pathway confers acquired gefitinib resistance in NSCLC cells via GJIC-independent induction of EMT
Source: Cell Death Dis. 2015 Jul 23;6(7):e1829–. doi: 10.1038/cddis.2015.197 (PMC4650742; doi:10.1038/cddis.2015.197)
Supplement: Supplementary Materials [file cddis2015197x1.doc]

**Supplementary Materials**

**Materials and methods**

*Cell Culture*

Human foreskin fibroblasts (HFFs) from adult human foreskin were prepared and maintained as previously described with informed consent [1-2].

**Supplementary Figure legends**

Supplementary Figure 1. RA (20 uM, 24 h) increased the GJIC of human foreskin fibroblasts (HFFs), as shown by parachute assay. Top, fluorescence images. Bottom, overlaid the corresponding phase-contrast images. Original magnification, ×200.

**References**

1. Aasen, T. and J.C. Izpisua Belmonte, *Isolation and cultivation of human keratinocytes from skin or plucked hair for the generation of induced pluripotent stem cells.* Nat Protoc, 2010. **5**(2): p. 371-82.

2. Ke, Q., et al., *Connexin 43 is involved in the generation of human-induced pluripotent stem cells.* Hum Mol Genet, 2013. **22**(11): p. 2221-33.
