# Supplementary figures and images for: Reciprocal positive regulation between Cx26 and PI3K/Akt pathway confers acquired gefitinib resistance in NSCLC cells via GJIC-independent induction of EMT
Source: Cell Death Dis. 2015 Jul 23;6(7):e1829–. doi: 10.1038/cddis.2015.197 (PMC4650742; doi:10.1038/cddis.2015.197)

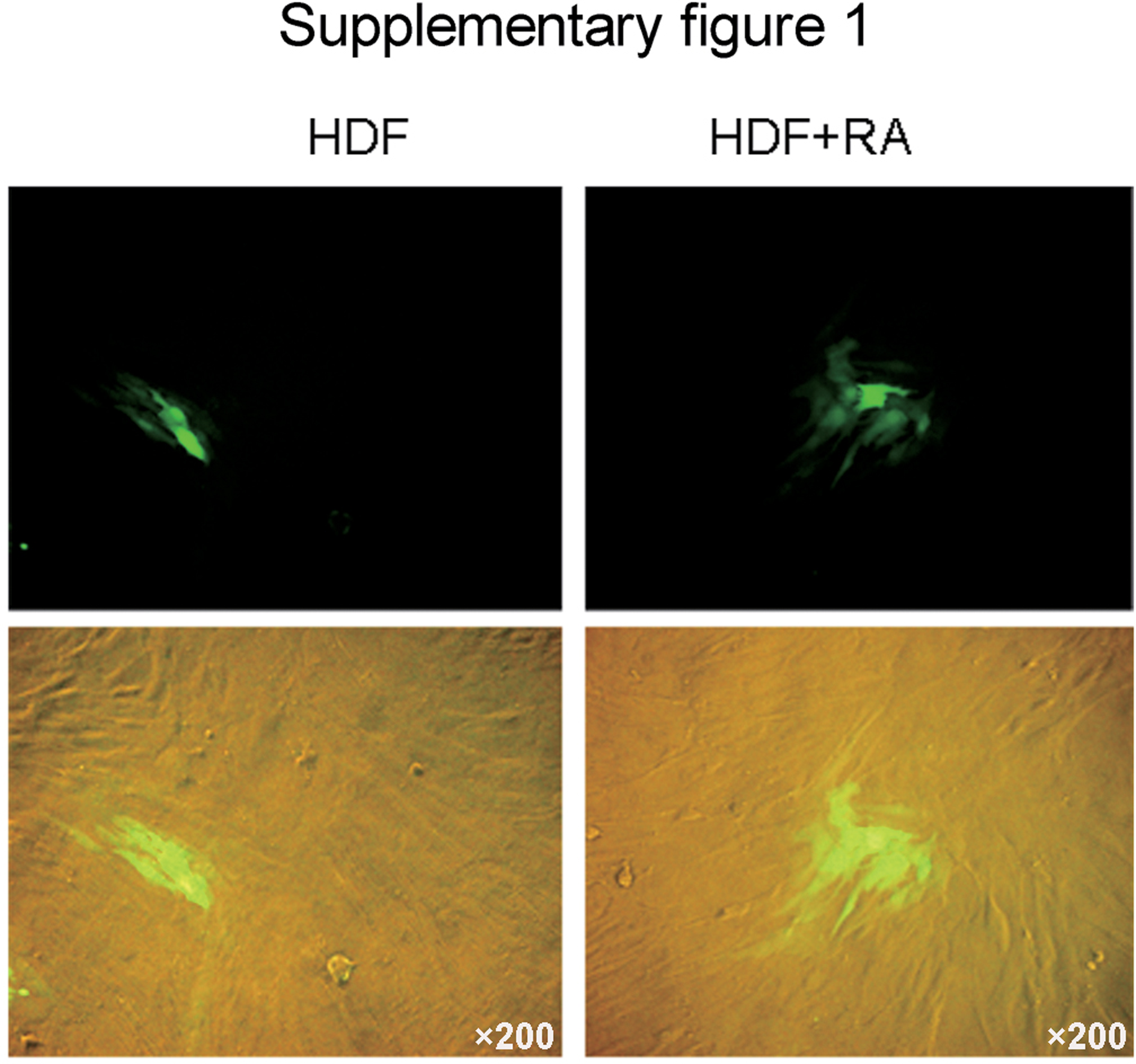

Supplement: Supplementary Figure 1 [file cddis2015197x2.tif]
